# First Insight into the Genome Sequence of *Clostridium thermobutyricum* DSM 4928, a Butyrate-Producing Moderate Thermophile

**DOI:** 10.1128/genomeA.00367-17

**Published:** 2017-05-18

**Authors:** Anja Poehlein, Abirami Anbalagan, Alexandra Nagel, Rolf Daniel

**Affiliations:** a Genomic and Applied Microbiology & Göttingen Genomics Laboratory, Institute of Microbiology and Genetics, Georg-August University of Göttingen, Göttingen, Germany; b Members of the Applied Bioinformatics in Microbiology Course of the Microbiology and Biochemistry M.Sc./Ph.D. program, Georg-August University of Göttingen, Göttingen, Germany

## Abstract

The moderately thermophilic and Gram-positive bacterium *Clostridium thermobutyricum* is strictly anaerobic and forms subterminally located endospores. It was isolated from horse manure compost. *C. thermobutyricum* produces butyrate as the main fermentation product. The draft genome consists of one circular chromosome (3.425 Mb) and contains 3,201 predicted protein-coding genes.

## GENOME ANNOUNCEMENT

*Clostridium thermobutyricum* is an anaerobic, moderately thermophilic, and Gram-positive bacterium with straight to slightly curved rod-shaped cells. The strain *C. thermobutyricum* DSM 4928 was isolated from horse manure samples collected from horse stables (1). The cells are 0.9 to 1.1 µm in diameter and 2.0 to 4.5 µm in length. *C. thermobutyricum* grows at temperatures ranging from 26 to 61.5°C, with an optimal temperature of approximately 55°C. It is able to utilize different substrates, including yeast extract, cellobiose, glucose, pyruvate, and d-glucoronic acid. *C. thermobutyricum* produces butyrate as the major fermentation product (1).

For DNA isolation *of C. thermobutyricum* DSM 4928, the MasterPure complete DNA purification kit was used as recommended by the manufacturer (Epicentre, Madison, WI, USA). The extracted DNA was employed to generate Illumina paired-end sequencing libraries, as recommended by the manufacturer (Illumina, San Diego, CA, USA). Sequencing was performed with a MiSeq instrument and the MiSeq reagent kit version 3, according to the protocols of the manufacturer (Illumina). Quality trimming was conducted using Trimmomatic version 0.3.6 (2) and resulted in 2,072,576 paired-end reads. Genome sequence assembly with SPAdes version 3.10.0 (3) yielded 111 contigs (>500 bp), with an average coverage of 113-fold. Validations of the assembly and read coverage were determined with Qualimap version 2.1 (4). The draft genome of *C. thermobutyricum* consists of one circular chromosome (3.425 Mb) exhibiting a G+C content of 26.43%. Automatic gene prediction and identification of rRNA and tRNA genes were performed using the software tool Prokka (5). The draft genome contains 14 rRNA genes, 70 tRNA genes, and 1 transfer-messenger RNA (tmRNA) gene. Gene prediction resulted in 2,305 protein-coding genes with a predicted function and 896 genes encoding hypothetical proteins. Seventeen predicted protein-coding genes were associated with prophages, and 23 genes were associated with multidrug resistance.

*C. thermobutyricum* is known for producing butyrate as the major fermentation product (1). Accordingly, the genome harbors putative genes encoding enzymes associated with butyrate formation, such as thiolase, 3-hydroxybutyryl-coenzyme A (CoA) dehydrogenase, enoyl-CoA hydratase, acyl-CoA dehydrogenase, and butyrate kinase. It has been shown that *C. thermobutyricum* is able to utilize acetate as a cosubstrate for the production of butyrate via coenzyme A transferase (6). This was supported by the presence of a putative gene in the genome which encodes a similar enzyme called succinyl-CoA: the coenzyme A transferase gene (6, 7). In addition to butyrate, *C. thermobutyricum* also produces acetate and lactate as fermentation products. Correspondingly, genes encoding lactate dehydrogenase, phosphate acetyltransferase, and acetate kinase were found to be present.

### Accession number(s).

This whole-genome shotgun project has been deposited at DDBJ/EMBL/GenBank under the accession number LTAY00000000. The version described here is the first version, LTAY01000000.
